# MAPK and phenylpropanoid metabolism pathways involved in regulating the resistance of upland cotton plants to *Verticillium dahliae*

**DOI:** 10.3389/fpls.2024.1451985

**Published:** 2024-09-24

**Authors:** Mingli Zhang, Yanjun Ma, Yuan Wang, Haifeng Gao, Sifeng Zhao, Yu Yu, Xuekun Zhang, Hui Xi

**Affiliations:** ^1^ Open Research Fund of Key Laboratory of Integrated Pest Management on Crops in Northwestern Oasis, Ministry of Agriculture and Rural Affairs, Urumqi, Xinjiang, China; ^2^ Key Laboratory of Oasis Agricultural Pest Management and Plant Protection Resources Utilization, College of Agriculture, Shihezi University, Shihezi, Xinjiang, China; ^3^ Cotton Research Institute, Xinjiang Academy of Agricultural Reclamation Sciences, Shihezi, Xinjiang, China

**Keywords:** *Verticillium dahliae*, transcriptome, MAPK pathway, phenylpropanoid biosynthesis, cotton

## Abstract

**Introduction:**

*Verticillium dahliae* causes a serious decline in cotton yield and quality, posing a serious threat to the cotton industry. However, the mechanism of resistance to *V. dahliae* in cotton is still unclear, which limits the breeding of resistant cultivars.

**Methods:**

To analyze the defense mechanisms of cotton in response to *V. dahliae* infection, we compared the defense responses of two upland cotton cultivars from Xinjiang (JK1775, resistant; Z8,susceptible) using transcriptome sequencing at different infection stages.

**Results:**

The results revealed a significant differential expression of genes in the two cotton cultivars post *V. dahliae* infection, with the number of DEGs in JK1775 being higher than that in Z8 at different infection stages of *V. dahliae*. Interestingly, the DEGs of both JK1775 and Z8 were enriched in the MAPK signaling pathway in the early and late stages of infection. Importantly, the upregulated DEGs in both cultivars were significantly enriched in all stages of the phenylpropanoid metabolic pathway. Some of these DEGs were involved in the regulation of lignin and coumarin biosynthesis, which may be one of the key factors contributing to the resistance of upland cotton cultivars to *V. dahliae* in Xinjiang. Lignin staining experiments further showed that the lignin content increased in both resistant and susceptible varieties after inoculation with *V. dahliae*.

**Discussion:**

This study not only provides insights into the molecular mechanisms of resistance to Verticillium wilt in Xinjiang upland cotton but also offers important candidate gene resources for molecular breeding of resistance to Verticillium wilt in cotton.

## 1 Introduction

Verticillium wilt is one of the most serious and destructive plant diseases threatening cotton production worldwide (Shaban et al., 2018). The most effective way to prevent and control Verticillium wilt is to cultivate and plant resistant cotton cultivars (Gao et al., 2011). However, due to the lack of germplasm resources with high resistance to Verticillium wilt and the long cycle and high production costs of conventional breeding methods, progress in breeding cotton against Verticillium wilt has been slow (Zhu et al., 2023). Studying the resistance and regulatory mechanisms of cotton to *V. dahliae* is crucial for breeding disease-resistant cotton. Understanding the response mechanisms of different disease-resistant cotton strains to *V. dahliae* will help clarify the interaction between cotton and *V. dahliae* and provide new insights for breeding disease-resistant cotton.

During fungal infection, the pattern recognition receptors on plant cell membranes recognize chitin oligosaccharides produced by chitin on fungal cell walls, triggering immune responses (Khajavian et al., 2022). *Gbvdr5*, a *Ve* homologous gene, recognizes the effector Ave1, promoting callose deposition, upregulating the expression of the defense-related genes *PR1* and *PR5*, and triggering programmed cell death to resist *V. dahliae* infection (Yang et al., 2018). MAPK cascades are involved in signaling multiple defense responses, including the biosynthesis/signaling of plant stress/defense hormones, reactive oxygen species (ROS) generation, stomatal closure, defense gene activation, phytoalexin biosynthesis, cell wall strengthening, and hypersensitive response (HR) cell death (Meng and Zhang, 2013). *GhPLDδ*, a plant phospholipase D (PLD) gene identified in upland cotton, enhances cotton resistance to *V. dahliae* by activating reactive oxygen species, MAPK cascades, and jasmonic and salicylic acid signaling pathways (Zhu et al., 2022). The GhMKK2-GhNTF6-GhMYC2 pathway, mediated by GhMKK2, induces the expression of flavonoid biosynthesis-related genes and increases the accumulation of flavonoids, thereby enhancing the cotton resistance to *Fusarium oxysporum* (Wang et al., 2022). The GhMPK9-GhRAF391-GhWRKY40a module upregulates *PR* genes mediated by *GhERF1b* and positively modulates cotton resistance to Verticillium wilt (Mi et al., 2024). These studies demonstrate the significant role of MAPK cascades in the resistance of cotton plants to pathogens.

Phenylpropanoid metabolites are essential for plant development and survival and play important roles in the defense against pathogen infection (Wang et al., 2019). Phenylalanine compounds are precursors of various phenolic compounds, such as flavonoids, isoflavones, and apigenin (Roberts et al., 2002). The accumulation of phenylalanine in plants can significantly reduce their sensitivity to pathogens (Kumar et al., 2020). *GhWRKY41* improves cotton resistance to *V. dahliae* by activating the expression of *GhC4H* and *Gh4CL*, promoting the accumulation of lignin and flavonoids (Xiao et al., 2023). *GhHSFA4* contributes positively to cotton resistance by mediating the secondary metabolic pathways of flavonoids and terpenoids, as well as the biosynthesis and signaling pathways of jasmonic acid (JA) (Liu et al., 2022). *GhWATs* play an important role in cotton defense against *V. dahliae* by regulating the biosynthesis of JA and the deposition of lignin in the xylem (Tang et al., 2019). *Gh4CL30* protects against Verticillium wilt by regulating the content of lignin and phenolic compounds (Xiong et al., 2021). These findings indicate that phenylpropanoid metabolites play an important role in cotton defense against *V. dahliae* infection.

Transcriptome analysis is an effective technique for studying changes in gene expression across whole genome of plants and has been widely used to identify potential genes and molecular mechanisms involved in various biotic stress processes (Guo et al., 2014). A previous study revealed that more genes were upregulated in cotton in response to strongly virulent *V. dahliae* strain V991 than to hypovirulent *V. dahliae* strain 1cd3-2 (Qiu et al., 2024). This indicates that the methods of preventing *V. dahliae* in cotton are complex, and the major genes involved in resistance to Verticillium wilt in upland cotton have not yet been identified. To further elucidate the molecular mechanisms underlying the resistance of upland cotton plants to *V. dahliae* in Xinjiang, we compared the changes in gene expression between resistant and susceptible cotton plants at different time points after inoculation with strongly virulent *V. dahliae* strain via transcriptome analysis. This study helps elucidate the interaction mechanism between cotton and *V. dahliae* more clearly and provides potential functional genes for disease resistance breeding.

## 2 Results

### 2.1 Resistance identification of JK1775 and Z8 against *V. dahliae*

**Figure 1:**
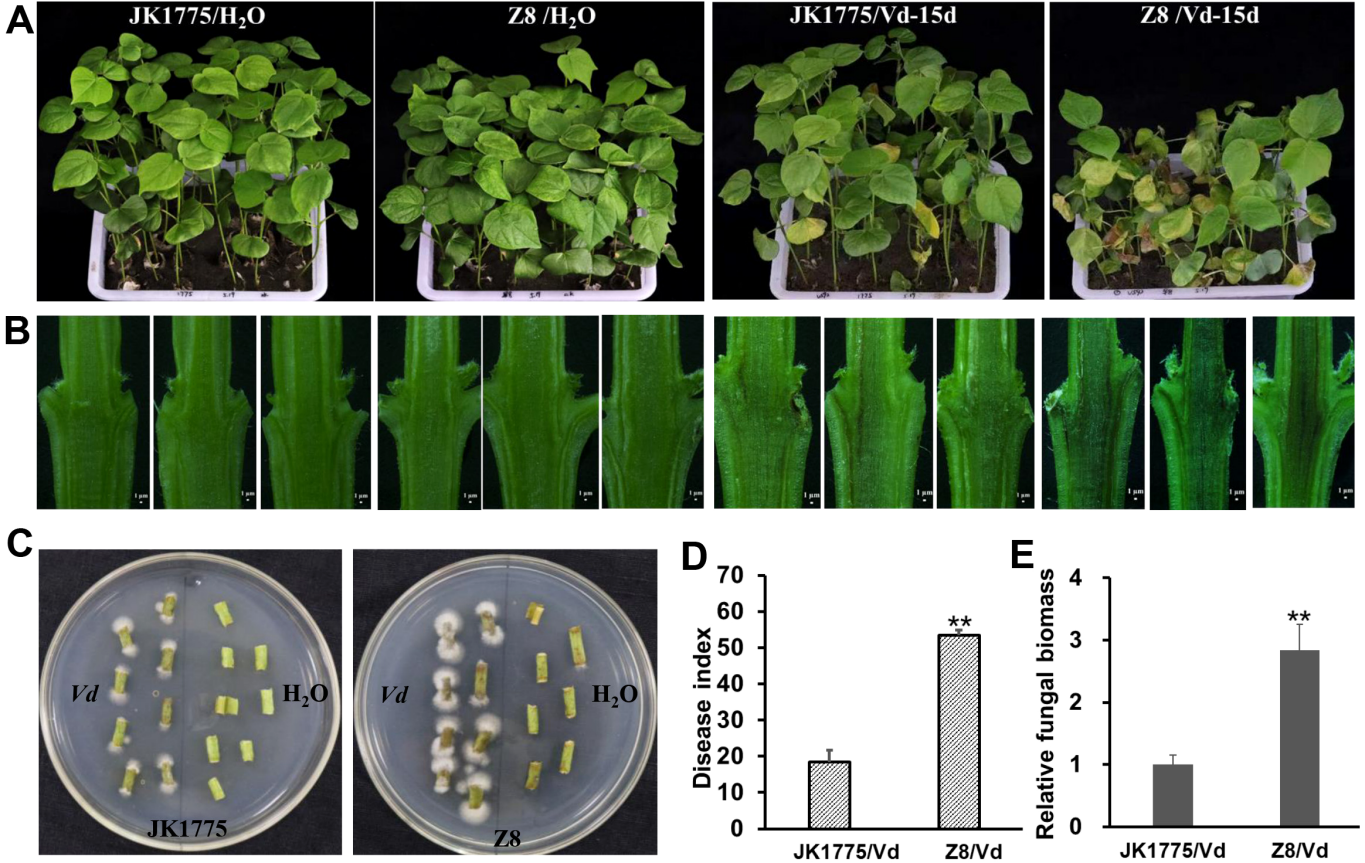
Different responses of JK1775 (R) and Z8 (S) to V592 at 15 dpi. **(A)** Disease symptoms of JK1775 and Z8 after inoculation with V592. **(B)** Observation of vascular bundle browning in longitudinal stem sections of JK1775 and Z8 plants cut lengthwise by hand. **(C)** Fungal recovery assay of the stems of JK1775 and Z8. **(D)** The disease indices of JK1775 and Z8. **(E)** Fungal biomass in the stems of JK1775 and Z8 plants was measured by qRT-PCR. Significance was determined using Duncan’s multiple range test, indicated by ** p ≤ 0.01.

The JK1775 and Z8 showed significantly different levels of resistance to *V. dahliae* in the field. To further confirm these results, we conducted a resistance identification assay in a greenhouse. 15 days after inoculation with the *V. dahliae* strain V592, more severe disease symptoms were observed in the Z8 plants than in the JK1775 plants (**Figure 1A**). The degree of browning in the vascular bundle of stem’s longitudinal profile was higher in Z8 than in JK1775 (**Figure 1B**). Additionally, more pathogen colonization was recovered from the stems of Z8 than from those of JK1775 (**Figure 1C**). The disease index and fungal biomass detected in Z8 plants were also higher than those in JK1775 plants (**Figures 1D, E**), which was consistent with the observed phenotypes. These results suggest that JK1775 and Z8 can serve as resistant and susceptible cultivars, respectively, for analyzing the mechanism of cotton resistance to Verticillium wilt.

### 2.2 RNA sequencing of resistant and susceptible upland cotton after *V. dahliae* infection

To explore the molecular response of upland cotton to *V. dahliae*, we obtained a transcriptome sequencing data from samples of JK1775 and Z8 at different stages after *V. dahliae* infection. A total of 1,603,445,914 clean reads were obtained, with the total number of raw and clean reads for each sample ranging from 46,895,158 to 59,805,106 and from 39,227,654 to 50,598,412, respectively (**Supplementary Table 1**). The Q20% and Q30% were higher than 99% and 96%, respectively (**Supplementary Table 1**). The GC content of these samples was between 44.08% and 45.15%, with an average GC content of 44.39% (**Supplementary Table 1**), indicating high sequencing quality across all samples. Over 91.69% of the reads were uniquely mapped to the upland cotton genome. The distribution of reads showed significantly higher number of reads derived from coding regions compared to intergenic or intronic regions, indicating that most reads originated from mature mRNAs (**Supplementary Table 2**). These results confirm that the RNA-seq data used in this study are highly reliable.

### 2.3 Comparison of DEGs between resistant and susceptible cotton cultivars against *V. dahliae*

**Figure 2:**
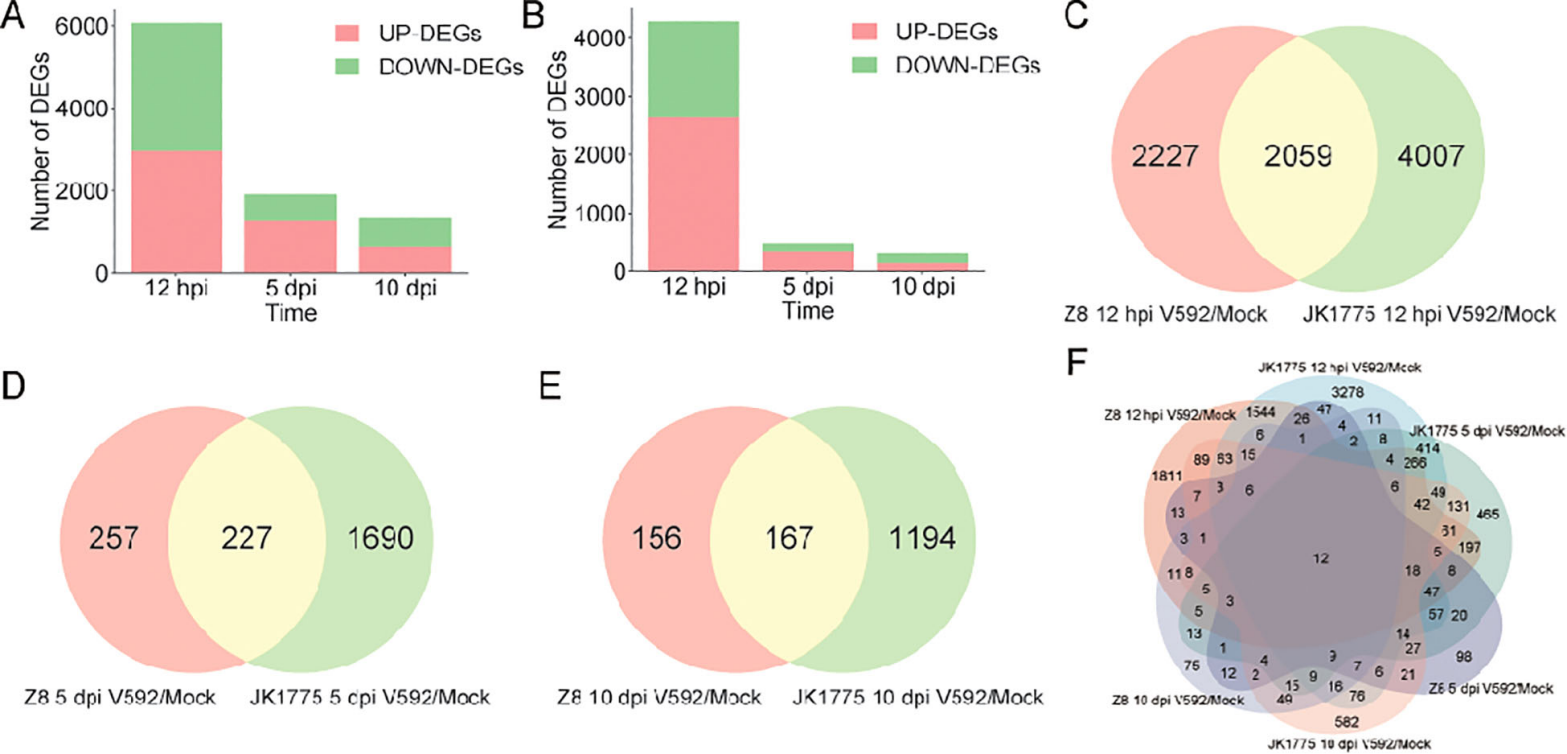
Comparison of the DEGs between JK1775 and Z8 after infection with V592 at different periods. Number of DEGs after Z8 **(A)** and JK1775 **(B)** were inoculated with V592 for different periods. Comparison of the DEGs between JK1775 and Z8 after infection with V592 at 12 hpi **(C)**, 5 dpi **(D)**, and 10 dpi **(E)**. **(F)** Comparison of DEGs between JK1775 and Z8 after infection with V592 at different time points via a Venn diagram.

To elucidate the resistance mechanism of upland cotton to *V. dahliae*, we analyzed the DEGs of JK1775 and Z8 at early (12 hpi), middle (5 dpi), and late (10 dpi) stages. Among the DEGs of the susceptible cultivar Z8, 2,642 genes were upregulated and 1,644 genes were downregulated at the early infection stage; 357 genes were upregulated and 127 genes were downregulated at the middle infection stage; and 142 genes were upregulated and 181 genes were downregulated at the late infection stage (**Figure 2A**). The number of DEGs in the resistant cultivar JK1775 was significantly higher than that in the Z8 cultivar. Among the DEGs of JK1775, 2,963 genes were upregulated and 3,103 genes were downregulated genes at the early infection stage; 1,306 upregulated genes and 611 downregulated genes at the middle infection stage; and 652 upregulated genes and 709 downregulated genes at the late infection stage (**Figure 2B**). There were 2,059, 227, and 167 DEGs in both cotton cultivars that responded to *V. dahliae* infection during the early, middle, and late infection periods, respectively (**Figures 2C–E**). Further analysis revealed that majority of DEGs were expressed during early infection stage, which may be critical for cotton resistance to *V. dahliae*. Importantly, the resistant cotton cultivar JK1775 mobilized more genes to combat *V. dahliae* infection than the susceptible cultivar Z8. Additionally, twelve genes were expressed at all stages of *V. dahliae* infection in both JK1775 and Z8 cultivars. (**Figure 2F**), suggesting that these may be key genes involved in cotton resistance to Verticillium wilt.

### 2.4 Identification of resistance-related pathways in resistant cultivars via KEGG analysis

**Figure 3:**
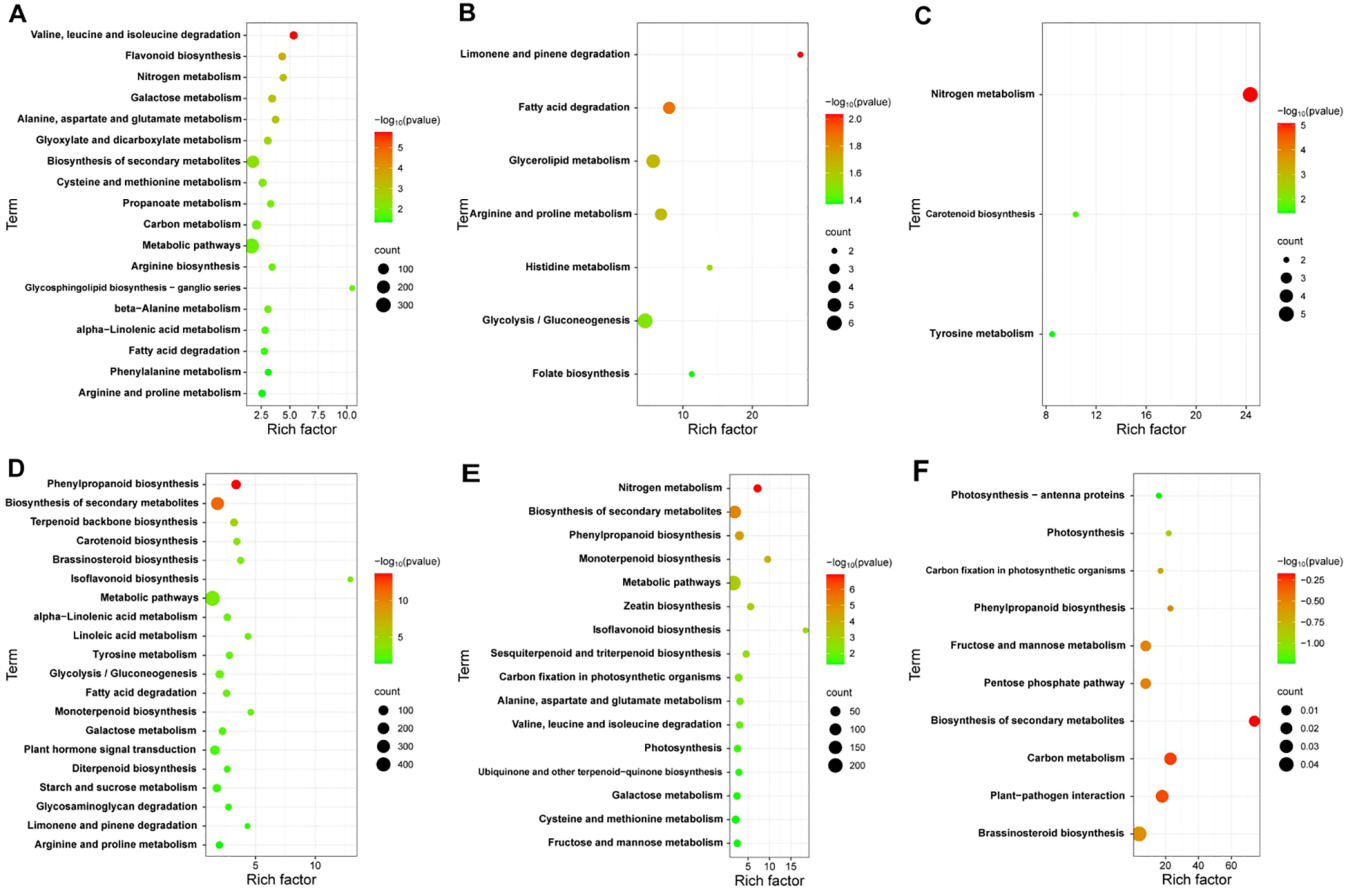
KEGG pathway analysis of DEGs in JK1775 and Z8 after infection with V592. KEGG significantly enriched DEGs in Z8 inoculated with V592 at 12 hpi **(A)**, 5 dpi **(B)** and 10 dpi **(C)**. KEGG significantly enriched DEGs in JK1775 inoculated with V592 at 12 hpi **(D)**, 5 dpi **(E)** and 10 dpi **(F)**.

To explore the key pathways involved in the response of cotton plants to *V. dahliae* infection, we analyzed the KEGG annotations of the DEGs from JK1775 and Z8 plants at different infection stages. The results revealed significant differences in the enrichment pathways of specific induced genes between the two cotton cultivars. In the early infection stage, the specific DEGs in Z8 were mainly involved in flavonoid biosynthesis, nitrogen metabolism, glyoxylic acid and dicarboxylic acid metabolism, cysteine and methionine metabolism, phenylalanine metabolism, and other pathways. In contrast, the DEGs of JK1775 that were specifically expressed in the early infection stage were related to phenylpropionic biosynthesis, terpenoid skeleton biosynthesis, carotenoid biosynthesis, brassinosteroid biosynthesis, isoflavone biosynthesis, linoleic acid metabolism, tyrosine metabolism, plant hormone signal transduction, and other pathways (**Figures 3A, D**). In the middle infection stage, the specific DEGs in Z8 were mainly involved in limonene and pinene degradation, fatty acid degradation, glycerolipid metabolism, arginine and proline metabolism, histidine metabolism, glycolysis/gluconeogenesis, and folic acid biosynthesis. In contrast, the DEGs in JK1775 that were specifically expressed in the middle stage were related to the biosynthesis of secondary metabolites, monoterpene biosynthesis, metabolic pathways, zeatin biosynthesis, isoflavone biosynthesis, and sesquiterpene and triterpene biosynthesis (**Figures 3B, E**). In the late stage of infection, the specific DEGs in Z8 were mainly involved in carotenoid biosynthesis and tyrosine metabolism, while the DEGs in JK1775 that were specifically expressed in the late stage were mainly involved in photosynthesis antenna protein, photosynthesis, carbon fixation of photosynthetic organisms, phenylpropionic acid biosynthesis, plant-pathogen interactions, brassinosteroid biosynthesis, and other pathways (**Figures 3C, F**). These findings highlight the complex and distinct response mechanisms of the two cotton cultivars to *V. dahliae* infection, and the resistant variety JK1775 has more pathways than does the susceptible variety Z8 in the middle and late stages of infection.

### 2.5 Response of plants to *V. dahliae* via the MAPK signaling pathway in cotton

**Figure 4:**
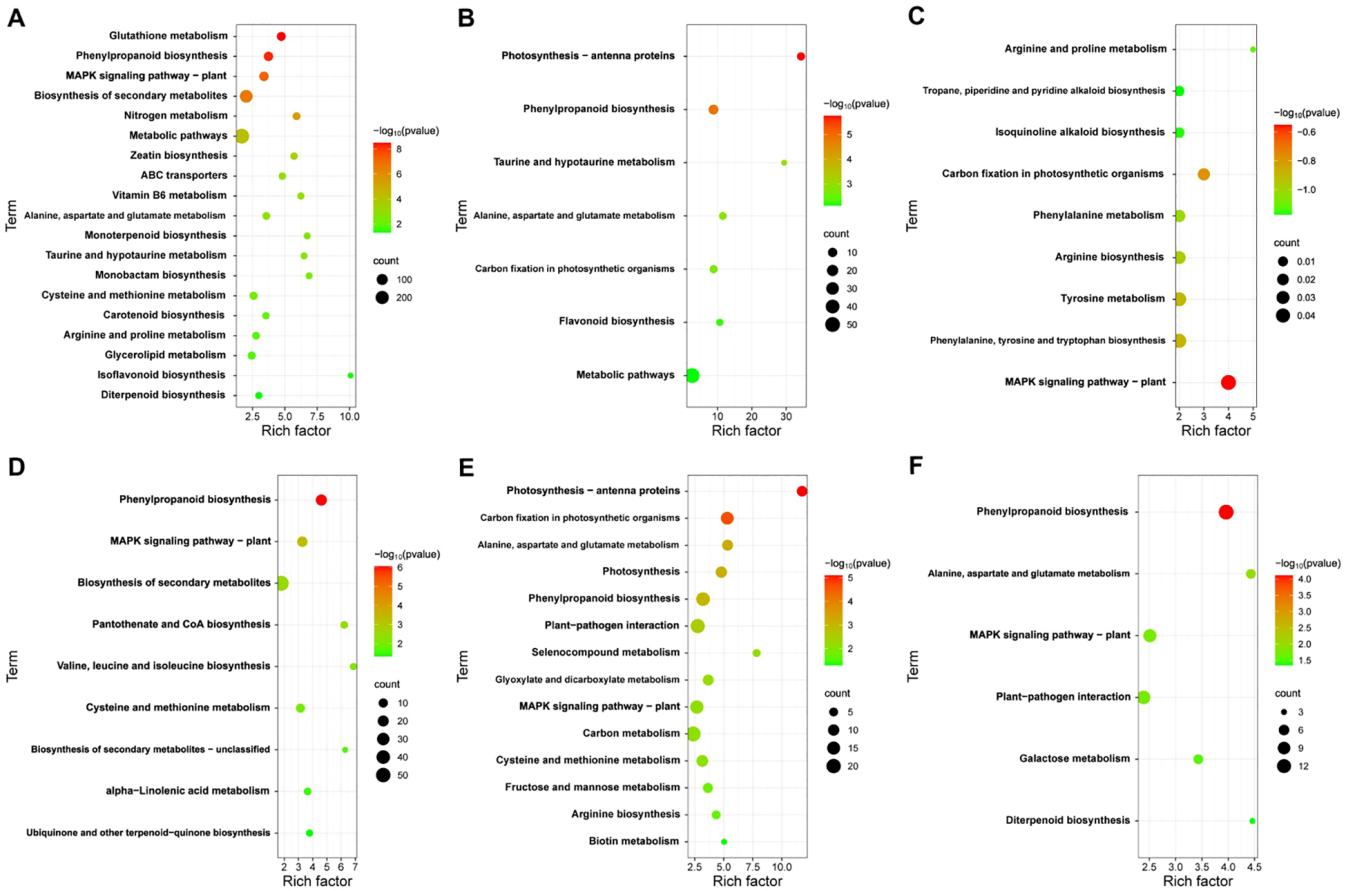
KEGG analysis of specific DEGs in JK1775 and Z8 post-V592 inoculation at 12 hpi **(A)**, 5 dpi **(B)** and 10 dpi **(C)**. KEGG enrichment analysis of genes significantly upregulated by JK1775 at 12 hpi **(D)**, 5 dpi **(E)** and 10 dpi **(F)** after *V. dahliae* inoculation compared with those of Z8.

To elucidate the function of DEGs at different time points in JK1775 and Z8 after *V. dahliae* inoculation, KEGG annotations of these genes were analyzed. In the early stage of infection, DEGs between Z8 and JK1775 were mainly enriched in glutathione metabolism, MAPK signaling pathway-plant, zeatin biosynthesis, ABC transporters, vitamin B6 metabolism, monolactam biosynthesis, and taurine and hypotaurine metabolism (**Figure 4A**). In the middle stage of infection, DEGs between Z8 and JK1775 were mainly enriched in photosynthetic antenna proteins, taurine and hypotaurine metabolism, carbon fixation of photosynthetic organisms, phenylpropanoid biosynthesis, and flavonoid biosynthesis (**Figure 4B**). In the late stage of infection, DEGs between Z8 and JK1775 were mainly enriched in MAPK signaling pathway-plant, arginine and proline metabolism, tropane, piperidine and pyridine alkaloid biosynthesis, isoquinoline alkaloid biosynthesis, carbon fixation of photosynthetic organisms, phenylalanine metabolism, arginine biosynthesis, tyrosine metabolism, and phenylalanine, tyrosine, and tryptophan biosynthesis (**Figure 4C**). Compared with those in Z8, the genes whose expression was upregulated in JK1775 were enriched in phenylpropanoid biosynthesis, the MAPK signaling pathway-plant, and the biosynthesis of secondary metabolites in the early stage of infection (**Figure 4D**). In the middle stage of infection, the genes upregulated in JK1775 were enriched in phenylpropanoid biosynthesis, plant-pathogen interactions, the MAPK signaling pathway-plant, and other pathways (**Figure 4E**). In the late stage of infection, the genes upregulated in JK1775 were enriched in phenylpropanoid biosynthesis, the MAPK signaling pathway, and plant−pathogen interactions (**Figure 4F**). In these three stages, the genes whose expression increased in JK1775 plants were enriched in phenylpropanoid biosynthesis and the MAPK signaling pathway. These results support the potential involvement of the MAPK signaling pathway and phenylpropionate biosynthesis in the response of cotton to *V. dahliae*.

### 2.6 Expression analysis of phenylpropanoid metabolic pathway genes

**Figure 5:**
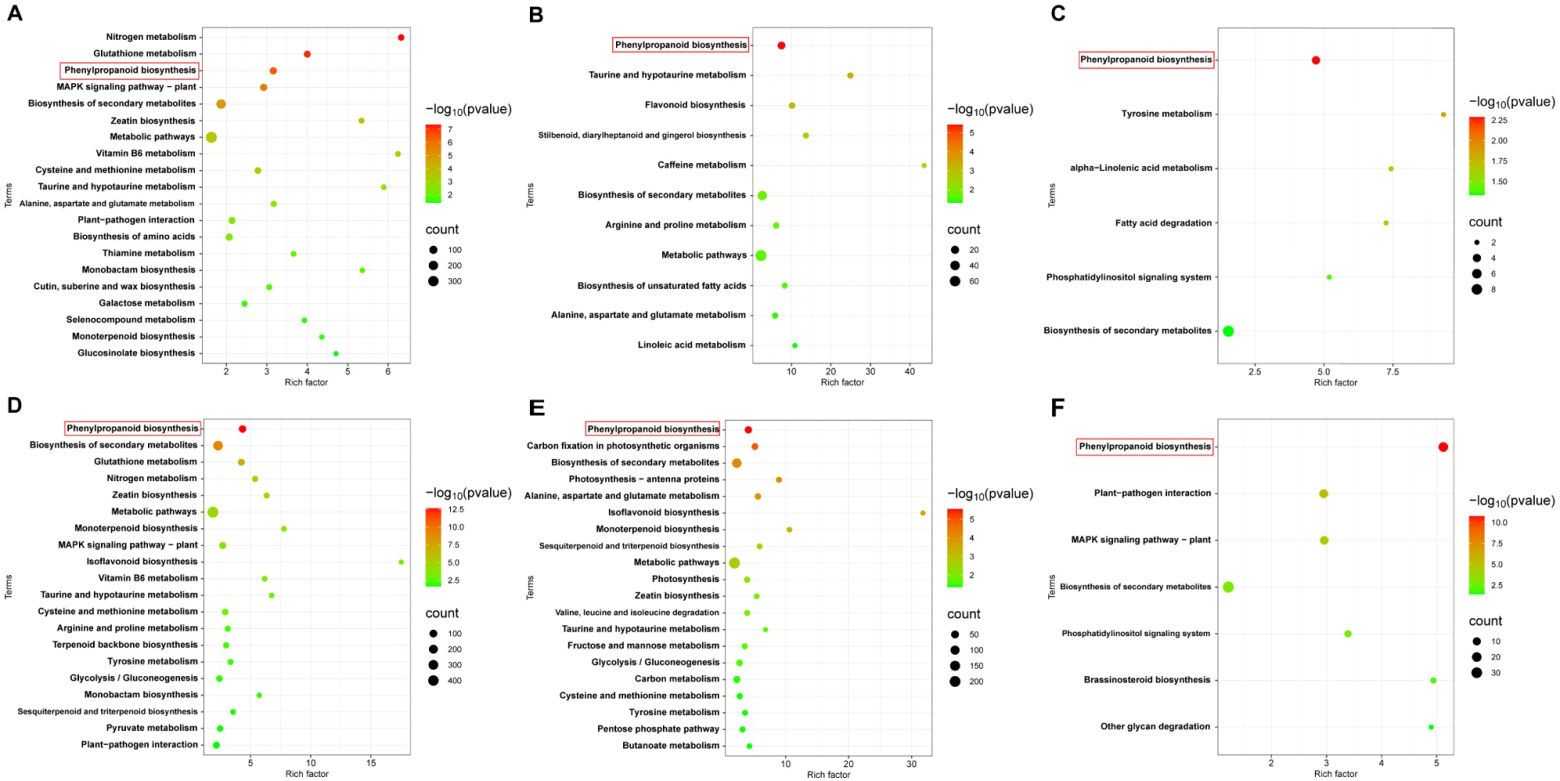
KEGG enrichment analysis of differentially upregulated genes. KEGG enrichment analysis of differentially upregulated genes in Z8 at 12 hpi **(A)**, 5 dpi **(B)** and 10 dpi **(C)**. KEGG enrichment analysis of differentially upregulated genes DEGs in JK1775 at 12 hpi **(D)**, 5 dpi **(E)** and 10 dpi **(F)**.

Coincidentally, both Z8 and JK1775 were inoculated with *V. dahliae*, and the upregulated genes involved in phenylpropanoid biosynthesis were significantly more enriched in inoculated plants than those not inoculated. At all three infection stages, the genes in the phenylpropanoid biosynthesis pathway were significantly upregulated (**Figure 5**). These results suggest that phenylpropanoid biosynthesis may be related to the induced resistance of cotton to *V. dahliae*.

**Figure 6:**
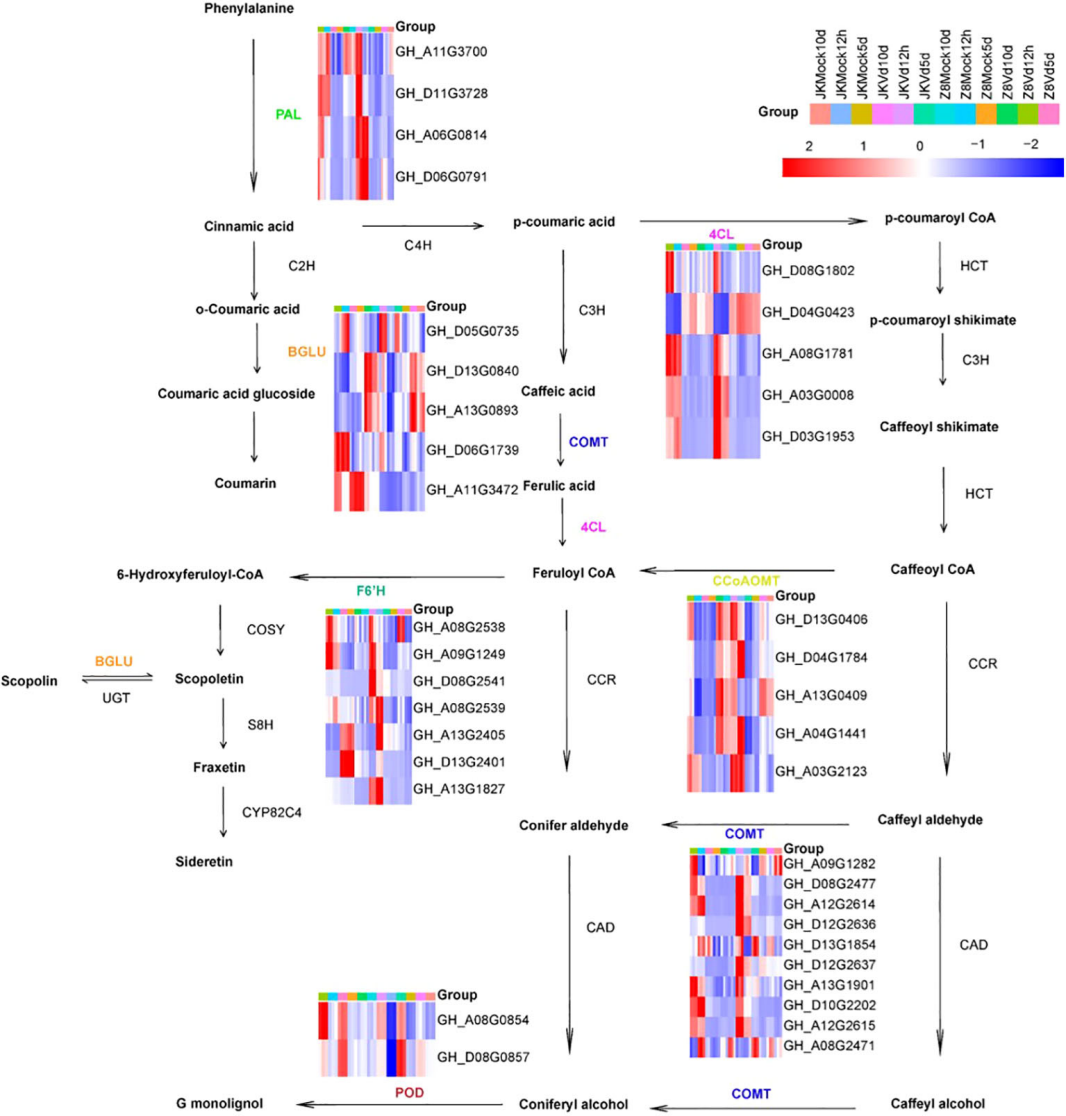
Expression analysis of DEGs involved in the phenylpropanoid pathway. The heatmap shows the expression of DEGs enriched in the phenylpropanoid biosynthesis pathway at 12 hpi, 5 dpi and 10 dpi after inoculation with JK1775 and Z8. Blue to red indicate gene expression levels from low to high, respectively. The expression level of each gene was estimated using log2 (fold change), and the color intensity was related to the fold change level. The cotton transcriptome data are shown in the figure. Enzyme abbreviations: PAL, phenylalanine ammonia lyase; C4H, cinnamate 4-hydroxylase; 4CL, 4-coumarate: CoA ligase; HCT, p-hydroxycinnamoyltransferase; C3H, 4-coumarate hydroxylase; CCoAOMT, caffeoyl-CoA-O-methyltransferase; CCR, cinnamoyl-CoA reductase; COMT, caffeic acid O-methyl transferase; CAD, cinnamyl alcohol dehydrogenase; BGLU, betaglucosidase; POD, peroxidase; F6’H, feruloyl-CoA 6’ hydroxylase1; C2H, cinnamic acid 2-hydroxylase.

Subsequently, we analyzed the differentially upregulated genes involved in phenylpropanoid pathways. DEGs related to phenylpropanoid biosynthesis were classified into seven gene families: four PAL, five 4CL, five CCoAOMT, ten COMT, seven F6’H, five BGLU, and two POD. These genes play crucial roles in the lignin and coumarin biosynthesis pathways (**Figure 6**). To further verify the accuracy of RNA-Seq analysis, eight genes from the phenylpropanoid metabolic pathway were selected for real-time quantitative PCR (qRT-PCR). The qRT-PCR results were highly consistent with RNA-Seq data (**Supplementary Figure 1**). In Z8, CCoAOMT, PAL, 4CL, COMT, BGLU, and POD were upregulated only during the early stage of infection, while the F6’H gene was upregulated only in the late stage. In JK1775, F6’H and COMT were upregulated during the early and middle stages; POD, BGLU, and 4CL were upregulated during the early and middle stages; and CCoAOMT and PAL were upregulated during the early and late stages of infection.

**Figure 7:**
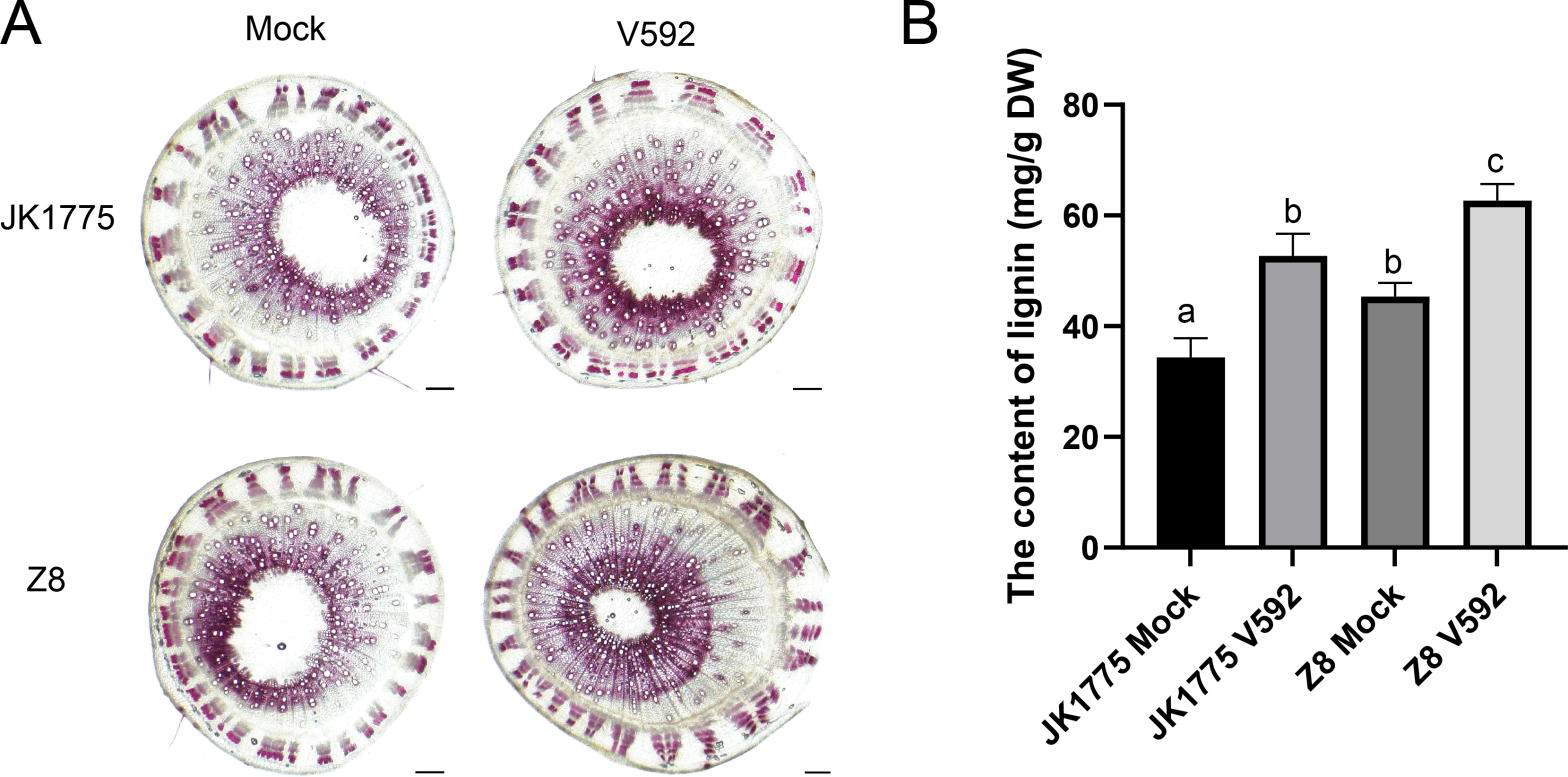
Lignin determination of JK1775 and Z8 at the 15 days after *V. dahliae* infection. **(A)** The histochemical analysis to observe the lignin deposition in stem of JK1775 and Z8 under *V. dahliae* infection. **(B)** The determination of the lignin in in stem of JK1775 and Z8 under *V. dahliae* infection.

A variety of peroxidases related to lignin synthesis were also upregulated in both cotton varieties during the early and middle stages of *V. dahliae* infection. Subsequently, we performed lignin staining experiments. The results showed that JK1775 and Z8 inoculated with *V. dahliae* had deeper resorcinol hydrochloride staining, higher xylem development, smaller vascular bundle pith area, and more lignified cell layers (**Figure 7**). This indicates that the process of cotton resistance to Verticillium wilt involves phenylpropanoid metabolism.

## 3 Discussion

*V. dahilae* affects more than half of cotton acreage, and *G. hirsutum* is particularly susceptible to *V. dahliae.* This susceptibility leads to a significant decline in fiber quality and yield (Li et al., 2023). Since Verticillium wilt survives as a microsclerotia in soil for a long time and has a wide range of hosts, the management strategy for cotton Verticillium wilt has thus far primarily relied on breeding and cultivating resistant varieties (Wang et al., 2023). With the development of comparative transcriptome and functional genomics technologies, several genes involved in the cotton defense response have been identified. For example, the thioredoxin-encoding gene *GbNRX1* plays a crucial role in the homeostasis of apoplastic reactive oxygen species in *V. dahliae*-infected cotton (Li et al., 2016).

During *V. dahliae* infection, spore attachment is observed at 12 hpi, mycelial colonization of epidermal cells at 5 dpi, and vascular tissue infection at 10 dpi (Zhang et al., 2020). In this study, we used RNA-seq to compare the transcriptome changes of resistant and susceptible *G. hirsutum* cultivars after inoculation with *V. dahliae*. The number of DEGs in the resistant variety JK1775 was higher than that in the susceptible variety Z8 at the early, middle, and late stages of infection. As inoculation progressed, the number of DEGs in the two varieties tended to decrease, indicating that the resistance of cotton plants to Verticillium wilt was mainly concentrated in the early stage. In a previous transcriptome analysis of the cotton response to *V. dahliae* infection, KEGG enrichment analysis revealed significant enrichment in plant−pathogen interactions, phenylpropanoid biosynthesis, and plant hormone signal transduction (Feng et al., 2023).

Subsequently, we performed KEGG enrichment analysis of common DEGs between resistant and susceptible varieties and found that these genes were significantly enriched in flavonoid biosynthesis, the MAPK signaling pathway-plant, and tryptophan biosynthesis. Compared with those of the susceptible variety Z8, the upregulated genes in the resistant variety JK1775 were enriched in phenylpropanoid biosynthesis and the MAPK signaling pathway in plants in the early and middle stages of infection. The rapid activation of MAPK cascades usually relays and amplifies PTI/ETI-induced downstream signals, such as transcriptional recombination and hormone signaling (Zhang and Zhang, 2022). Previous studies have reported that silencing *GhMKK2*, *GhMKK4*, *GhMKK6*, *GhMKK9*, *GhMPK9*, *GhMPK13*, and *GhMPK25* impairs cotton resistance to *V. dahliae* infection, while silencing *GhMKK10* increases resistance to *V. dahliae* infection (Meng et al., 2018). The overexpression of *GbMPK3* in cotton activated SA signal transduction but reduced resistance to *V. dahliae* (Long et al., 2020). This may be because the MAPK signaling pathway in resistant varieties is more responsive to *V. dahliae* than in susceptible varieties, resulting in a higher number of DEGs in the resistant cultivar JK1775 than in the susceptible cultivar Z8.

In this study, we identified 37 DEGs involved in phenylpropanoid biosynthesis through KEGG enrichment analysis of the transcriptome. These DEGs were involved in the synthesis of various important proteins, including caffeoyl-CoA O-methyltransferase (CCoAOMT), phenylalanine-ammonia lyase (PAL), 4-hydroxycinnamoy-CoAligase (4CL), caffeic acid 3-O-methyltransferase (COMT), BGLU (β-glucosidase), feruloyl CoA ortho-hydroxylase (F6’H), and peroxidase (POD) (**Figure 6**). After inoculation with *V.dahliae*, lignin content increased in both resistant and susceptible varieties; increased, however, the increase was more pronounced in the susceptible varieties. This may be due to the presence of multiple disease-resistant metabolic pathways (e.g., MAPK-plant, plant-pathogen interaction) in resistant varieties, whereas susceptible varieties have fewer such pathways, resulting in a lower increase in lignin (**Figure 7**). 4-Coumaric acid coenzyme A ligase is a key enzyme in the phenylpropanoid pathway. Treatment of blueberries with methyl jasmonate (MeJA) induces 4-coumaric acid coenzyme A ligase activity, thereby enhancing plant disease resistance (Wang et al., 2020). Caffeic acid O methyltransferases (COMTs) are crucial for lignin synthesis. *GhCOMT28*, *GhCOMT39*, *GhCOMT55*, *GhCOMT56*, and *GhCOMT57* respond to Verticillium wilt, potentially contributing to t resistance through lignin synthesis. Overexpression of these genes in wheat has been shown to enhance resistance to *Rhizoctonia cerealis* (Wu et al., 2021). Scopoletin, a coumarin with antibacterial properties is accumulated in response to F6’H gene (Rutul et al., 2021; Siwinska et al., 2014). These findings suggest that phenylpropanoid metabolites play an important role in plant defense responses against pathogens.

## 4 Materials and methods

### 4.1 Plant materials and treatments

Two cotton cultivars were used in this study: *Gossypium hirsutum* cv. Jinken 1775 (JK1775, resistant to Verticillium wilt) and Xinluzao 8 (Z8, susceptible to Verticillium wilt). These cultivars were provided by the Cotton Research Institute, Xinjiang Academy of Agricultural Reclamation Sciences. After soaking the cotton seeds for germination, the seeds were planted in sterile vermiculite for 3 days. The roots were then cleaned with tap water, and the plants were transplanted to Hoagland’s nutrient solution under greenhouse conditions (16 h/8 h light/dark, 25-28°C).

### 4.2 Fungal pathogen inoculation assay and recovery assay

V592 is a highly aggressive *V. dahliae* isolate that belongs to the defoliating pathotype (Wang et al., 2020). Initially, the V592 strain was cultured on potato dextrose agar (PDA) plates for 3 days at 25°C and then transferred to Czapek Dox liquid media with shaking for 3 days at 180 rpm and 25°C. The cotton seedlings (2 weeks old) were inoculated with a spore suspension (1×10^7^ spores/mL) by root dipping method (Wang et al., 2021). There were at least 15 seedlings per treatment, with three replicates. Roots were collected at different time points (12 hpi, 5 dpi, and 10 dpi) for RNA extraction after *V. dahliae* infection.

The disease level of the cotton leaves was classified from 0 to 4, and the disease index was used to evaluate the susceptibility of the cotton seedlings to *V. dahliae* according to the following formula: DI = [Σ (disease grades × number of diseased leaves)/(the number of total leaves×4)] × 100 (Gao et al., 2013). A fungal recovery assay from the stems was conducted, and the stems cut lengthwise by hand were observed under a stereoscopic microscope to confirm the colonization of pathogens at 14 dpi. Moreover, the stems above the cotyledons were collected for DNA extraction by CTAB method to measure fungal biomass via qRT-PCR (Gong et al., 2018). The fungus-specific ITS1-F primer (Fradin et al., 2011) was combined with the *V. dahliae*-specific reverse primer ST-VE1-R (Gong et al., 2018) to target the internal transcribed spacer region of the ribosomal DNA, and *GhUB7* was used as a reference gene (Gardes and Bruns, 1993). The fungal recovery assay was performed based on previously described methods (Zhang et al., 2011). After 4 days of culture, the colonies of rotifers were observed.

#### 4.2.1 RNA-sequencing analysis and qRT-PCR

An RNA extraction kit (Tiangen Biotech, China) was used to extract total RNA from cotton roots sampled at 12 hpi, 5 dpi, and 10 dpi according to the manufacturer’s protocol (Tiangen Biotech, China). After the total RNA of the samples was identified, the library was constructed, and then RNA-seq was performed based on the Illumina HiSeq 2500 high-throughput sequencing platform. Clean reads were obtained by removing reads containing connectors, low-quality reads, and reads containing poly-N from the original data. The Q20, Q30, and GC contents were calculated. Next, the clean reads were aligned to the genome of upland cotton TM-1.

The RPKM (reads per kilobase per million mapped reads) value was used as a measure of gene expression (Mortazavi et al., 2008). The RPKM method can eliminate the influence of gene length and sequencing depth on gene expression calculations, allowing for direct comparisons of gene expression differences between various samples. When a gene had multiple transcripts, the longest transcript was selected to calculate its sequencing coverage and expression. An absolute value of logFC > 1 and a P < 0.05 were used as the criteria. The results were analyzed by bioinformatics methods. Differentially expressed genes (DEGs) were obtained by comparative analysis of the unigenes. Kyoto Encyclopedia of Genes and Genomes (KEGG) pathway enrichment analysis was performed using KOBAS software. qRT-PCR was performed on a 7500 Real-Time PCR system with a reaction volume of 15 μL. Some DEGs were randomly selected for qRT-PCR verification to confirm the reliability of the RNA-seq data.

### 4.3 Visualization and quantification of lignin

The lignin content of cotton stalks was detected by phloroglucinol staining. The same parts of cotton stalk slices were soaked in 2% phloroglucinol solution for 10 min and then transferred to 18% hydrochloric acid solution for 5 min (Xiao et al., 2023). After the reaction, the samples were quickly taken out, observed under a microscope (DP74, Olympus), and photographed. The lignin-mercaptoacetic acid reaction was used to determine the lignin content in 5 mg of powder, based on previous studies (Xiao et al., 2023). Each experiment was repeated three times.

### 4.4 Statistical analysis

The qRT-PCR results were analyzed by the 2ΔCt method (Livak and Schmittgen, 2001) and graphed using GraphPad Prism v9.5. Significant differences were tested by using one-way ANOVA via SPSS v 17.0. The data are shown as the mean ± standard deviation. Statistically significant differences are indicated with an asterisk, *: P < 0.05, **: P < 0.01. A heatmap was generated with https://www.bioinformatics.com.cn, an online platform for data analysis and visualization (Tang et al., 2023).

## 5 Conclusions

**Figure 8:**
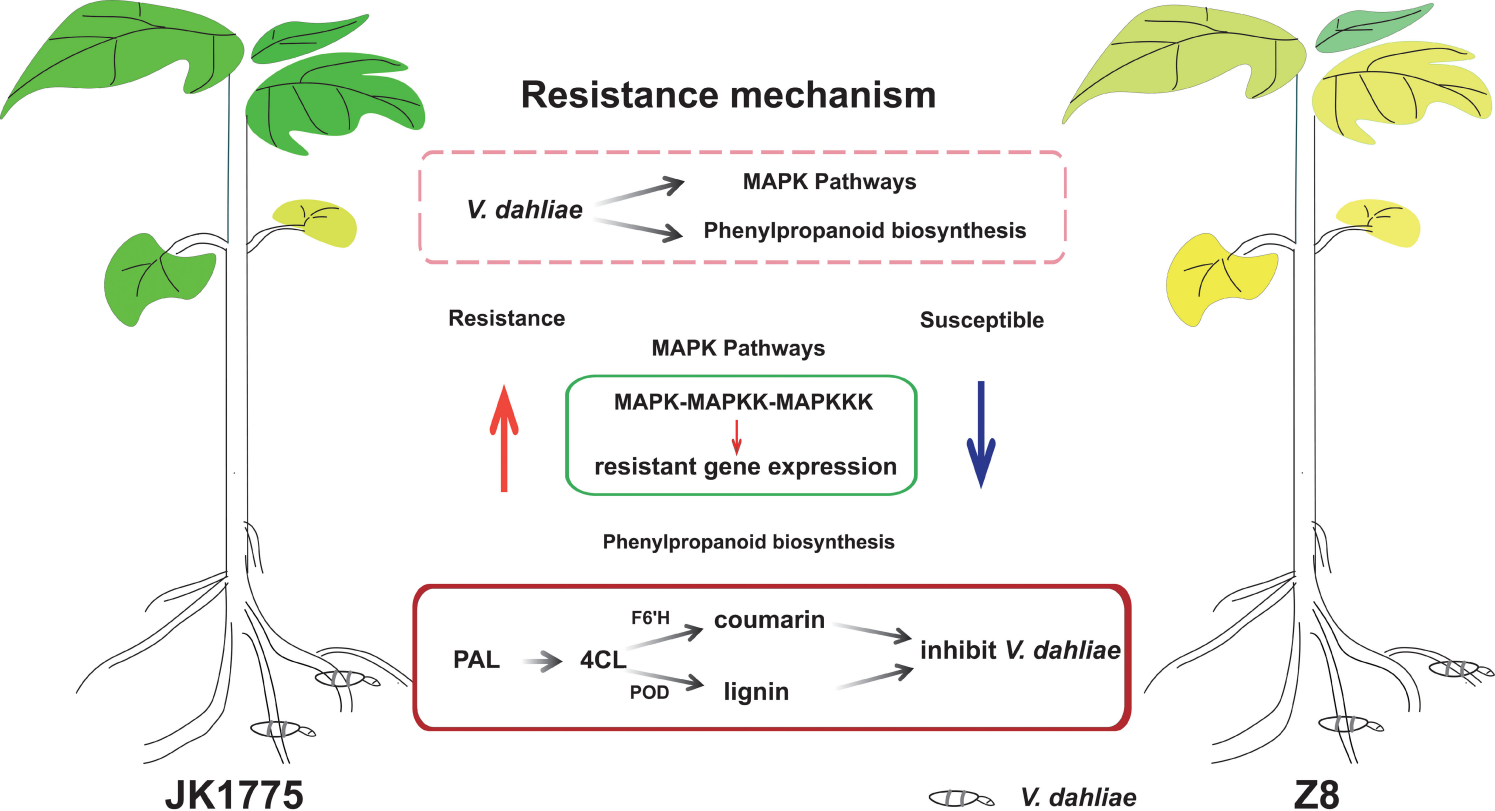
The proposed reaction mechanism of resistant and susceptible cotton to *V. dahliae* infection.

The transcriptome adaptability of resistant and susceptible cotton plants to *V. dahliae* infection was elucidated by high-throughput sequencing technology. KEGG pathway analysis revealed that the upregulated genes in resistant cultivars were significantly enriched in the MAPK signaling pathway compared with those in susceptible cultivars. Through KEGG analysis of the upregulated genes in the two cultivars, it was found that the genes in the phenylpropanoid metabolic pathway were upregulated in both cultivars. The encoding of F6’H during the transformation of phenylpropanoids to coumarin compounds may be the key to the resistance of local Xinjiang cotton varieties to Verticillium wilt (**Figure 8**). Our results have extremely important scientific research value and practical significance for the cultivation of cotton germplasm resistant to Verticillium wilt in Xinjiang.

## Data Availability

The sequenced raw reads generated in this study have been submitted to the National Center for Biotechnology Information (NCBI) with BioProject ID: PRJNA1121084.
