# Corrigendum: MAPK and phenylpropanoid metabolism pathways involved in regulating the resistance of upland cotton plants to *Verticillium dahliae*

**DOI:** 10.3389/fpls.2025.1607838

**Published:** 2025-04-25

**Authors:** Mingli Zhang, Yanjun Ma, Yuan Wang, Haifeng Gao, Sifeng Zhao, Yu Yu, Xuekun Zhang, Hui Xi

**Affiliations:** ^1^ Open Research Fund of Key Laboratory of Integrated Pest Management on Crops in Northwestern Oasis, Ministry of Agriculture and Rural Affairs, Urumqi, Xinjiang, China; ^2^ Key Laboratory of Oasis Agricultural Pest Management and Plant Protection Resources Utilization, College of Agriculture, Shihezi University, Shihezi, Xinjiang, China; ^3^ Cotton Research Institute, Xinjiang Academy of Agricultural Reclamation Sciences, Shihezi, Xinjiang, China

**Keywords:** *Verticillium dahliae*, transcriptome, MAPK pathway, phenylpropanoid biosynthesis, cotton

In the published article, there was an error in the **Funding** statement. Due to their own negligence, the number of some funded projects is repeated. Talent Innovation Science and Technology Team (2022TSYCTD0022) appeared twice: “The author(s) declare financial support was received for the research, authorship, and/or publication of this article. This research was funded by Shihezi University high-level talents research project(2022ZK015); Bingtuan Science and Technology Program (2022ZD056, 2023CB007-08); Open Research Fund of Key Laboratory of Integrated Pest Management on Crops in Northwestern Oasis, Ministry of Agriculture and Rural Affairs (KFJJ202305), Tianshan Talent Innovation Science and Technology Team (2022TSYCTD0022), Tianshan Talent Innovation Science and Technology Team(2022TSYCTD0022)”. The correct **Funding** statement appears below.

“The author(s) declare financial support was received for the research, authorship, and/or publication of this article. This research was funded by Shihezi University high-level talents research project(2022ZK015); Bingtuan Science and Technology Program (2022ZD056, 2023CB007-08); Open Research Fund of Key Laboratory of Integrated Pest Management on Crops in Northwestern Oasis, Ministry of Agriculture and Rural Affairs (KFJJ202305), Tianshan Talent Innovation Science and Technology Team (2022TSYCTD0022)”.

The authors apologize for this error and state that this does not change the scientific conclusions of the article in any way. The original article has been updated.

