# Supplementary figures and images for: MAPK and phenylpropanoid metabolism pathways involved in regulating the resistance of upland cotton plants to Verticillium dahliae
Source: Front Plant Sci. 2024 Sep 24;15:1451985. doi: 10.3389/fpls.2024.1451985 (PMC11458520; doi:10.3389/fpls.2024.1451985)

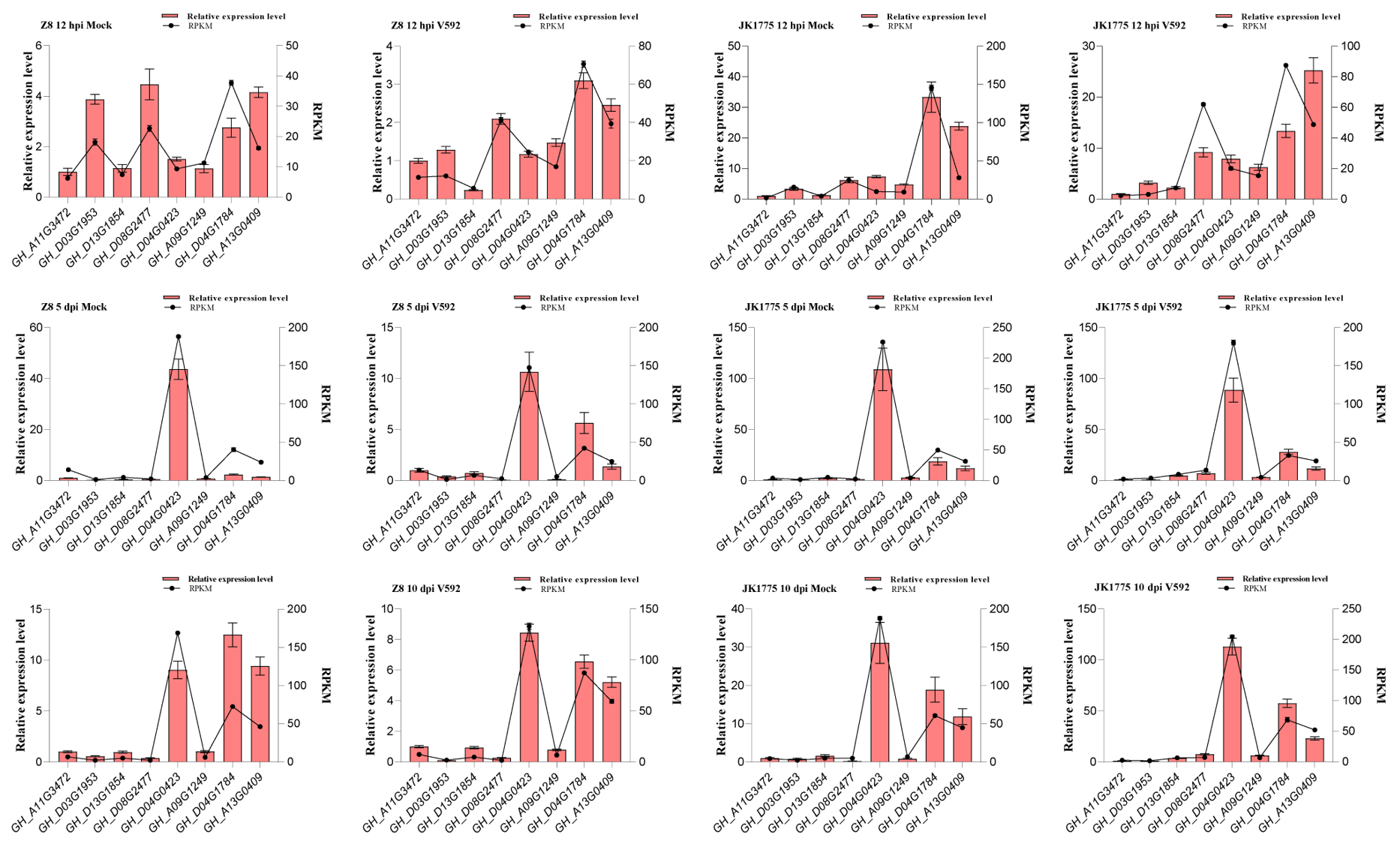

Supplement: Supplementary file 5 [file Image1.tif]
